# In Vitro Repair of Fractured Fiber-Reinforced Cusp-Replacing Composite Restorations

**DOI:** 10.1155/2011/165938

**Published:** 2011-09-15

**Authors:** Willem M. M. Fennis, Cees M. Kreulen, Arzu Tezvergil, Lippo V. J. Lassila, Pekka K. Vallittu, Nico H. J. Creugers

**Affiliations:** ^1^Department of Oral-Maxillofacial Surgery, Prosthodontics and Special Dental Care, University Medical Centre Utrecht, Utrecht, The Netherlands; ^2^Department of Oral Function and Prosthetic Dentistry, Radboud University Nijmegen Medical Centre, P.O. BOX 9101, 6500 HB Nijmegen, The Netherlands; ^3^Department of Prosthetic Dentistry and Biomaterials Research, Institute of Dentistry, University of Turku, Lemminkäisenkatu 2, 20520 Turku, Finland

## Abstract

*Objective*. To assess fracture resistance and failure mode of repaired fiber-reinforced composite (FRC) cusp-replacing restorations. *Methods*. Sixteen extracted human premolars with fractured cusp-replacing woven (Group (A)) or unidirectional (Group (B)) FRC restorations from a previous loading experiment were repaired with resin composite and loaded to fracture. *Results*. Differences in fracture loads between groups were not statistically significant (*P* = 0.34). Fracture loads of repaired specimens were significantly lower than those of original specimens (*P* = 0.02 for Group (A) and *P* < 0.001 for Group (B)). Majority of specimens showed failure along the repaired surface. In Group (B) 89% of specimens showed intact tooth substrate after restoration fracture, while this was 28% in Group (A) (*P* = 0.04). *Conclusion*. Fractured cusp-replacing FRC restorations that are repaired with resin composite show about half of fracture resistance of original restorations. Mode of failure with a base of unidirectional fibers is predominantly adhesive.

## 1. Introduction

An advantage of resin composite restoratives is the capacity to repair restorations by bonding new material to in vivo present resinous material [1–15]. This is a challenging approach compared to complete renewal of the restoration, especially with partial fracture of large restorations. It requires less investment, financially and biologically, to reconstruct the tooth by a bonded repair. A drawback is the reported lower interfacial bond strength of the two resin substrates as compared to the cohesive strength of the material due to the highly cross-linked nature of light-cured composite resins [1, 3, 5, 8].

One of the most attractive settings to investigate the benefits of repair is the resin composite restoration that replaces cusps [16–18]. This type of extensive plastic restoration is a tooth tissue saving intervention in case of loss of a cusp as an alternative to complete crown coverage. At present this restoration is primarily indicated in case of a fractured cusp with a premolar that includes a Class II amalgam or composite restoration. The restoration can be constructed with minimal tooth reduction, provided that sufficient sound dental hard tissue remains to adhere to.

Loading experiments with cusp-replacing particulate resin composite restorations frequently showed cohesive fractures of the material, followed by a destructive fracture of tooth substrate [19–21]. To prevent this type of crown-root fractures, a bonded resin-impregnated fiber layer can be applied to the cavity surface. This provides a stress-breaking shield comparable to the metal substructure of a porcelain-fused-to-metal crown. With the two cusps of a premolar covered by the restoration, the fiber layer may even prevent splitting of the root. Indeed, application of woven or unidirectional glass fiber-reinforced composite (FRC) had a beneficial effect on the mode of failure in laboratory loading tests [20, 22]. And after fracture, repair was expected to be feasible.

Generally, bonded repair of resin restorations can be achieved by mechanical and chemical surface treatment of the fractured restoration. The effects of roughening the surface with a diamond bur, air-particle abrasion, acid etching, and silane treatment have been investigated [1, 3, 5, 7, 9–12]. In case of repair of FRC restorations it was found that a combination of air-particle abrading and silane treatment provides optimal fracture resistance [3, 7, 9, 12].

With regard to repair of fractured cusp-replacing resin composite restorations with FRC basings no data are available yet. The objective of this study was therefore to assess these repaired restorations. A previous loading experiment did not reveal a difference in fracture resistance between cusp-replacing restorations with woven or with unidirectional FRCs [20]. Therefore it is hypothesized that such a difference is absent with repaired FRC restorations replacing cups. Furthermore, it is expected that fracture resistance of the repaired restorations is inferior to that of the original restorations and that fracture surfaces correspond to the repaired interface, without fracture of tooth substrate.

## 2. Materials and Methods

### 2.1. Specimens

**Figure 1:**
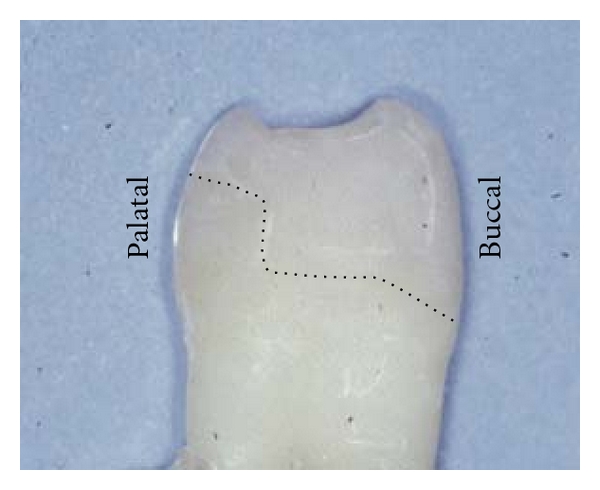
Upper premolar with original cusp-replacing resin composite restoration with FRC basing. Dotted line indicates cavity surface and position of fiber layer.

**Figure 2:**
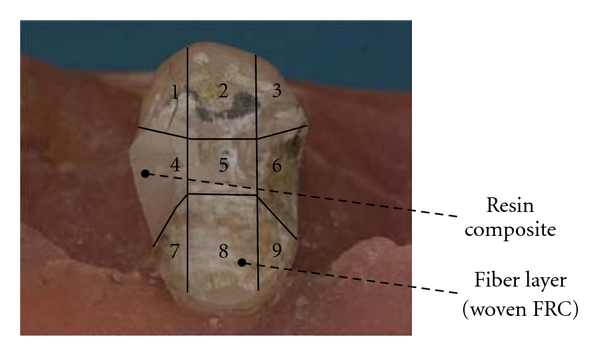
Example of specimen before repair with areas for repair surface evaluation. Surface areas 1–3 include the palatal cusp; 4 and 6 include the mesial/distal box; 5 includes the step; 7–9 include the buccal cusp.

Sixteen extracted human premolars with fractured cusp-replacing FRC restorations were used. These specimens resulted from a previous loading experiment with cusp-replacing FRC restorations [20]. The restorations included the buccal cusp, an MOD cavity, and the palatal cusp, which was reduced approximately 1.5 mm in height by preparation. Preparations were standardized by using a copy-milling procedure. In that study a resin-impregnated fiber layer was applied to the cavity surface before restoration, and two types of fiber reinforcement were used (Figure 1). In Group (A) two layers (thickness 0.06 mm per layer) of woven light polymerizable polymer-monomer gel-impregnated E-glass fibers (EverStickNet, StickTech, Turku, Finland) were used, in Group (B) one layer (0.2 mm) of continuous unidirectional light polymerizable polymer-monomer gel-impregnated E-glass fibers (EverStick, StickTech, Turku, Finland) with the fibers in buccal-palatal direction. Following the fiber layers, the specimens were built up with particulate resin composite (Clearfil Photo Posterior US, Kuraray, Osaka, Japan). These specimens are referred to as the “original” restorations. They were loaded until fracture and seven specimens in original Group (A) and nine in original Group (B) fractured in a way that repair of the restoration was considered possible (Figure 2). After the load test the premolars were still embedded in an acrylic block, they were stored in water for 21 months and used for the present study. In between the actual procedures of the experiment the specimens were also stored in water.

Before repair, the fracture surfaces of the specimens were visually evaluated for three characteristics: (1) presence of resin composite material, (2) presence of fiber layers, and (3) fibers being loose. To do so, the repair surface of each specimen was divided in nine areas (Figure 2), and each area was assessed. Assessment was based on a two-examiner agreement. The nine areas were grouped into four major restoration zones, and for each zone per group the status was described (Table 1). The bulk of the resin composite material was not present anymore for all of the restorations, while for the majority of the specimens of original Groups (A) and (B), the resin composite material and the fiber layers covering the palatal cusp were still present. The fiber layers at the occlusal step were present in all specimens. In the mesial and distal boxes and on the buccal cusp about 50–75% of the fiber layers was still present, with more fiber-reinforced material left in Group (B) than in Group (A). Loose fiber layers at the step, in the boxes, and at the buccal cusp were observed for about 10–45% of the fiber layers present. These loose fibers were observed more frequently in original Group (A) than in original Group (B).

### 2.2. Repair

Each specimen was sandblasted with Al_2_O_3_ (50 *μ*m, 3.2 bar, 10 seconds) and etched for 20 seconds using a 37% phosphoric acid etch-gel (Superlux-Thixo Etch, DMG, Hamburg, Germany). Subsequently, the fracture surface was rinsed thoroughly and air-dried gently. Dentin primer, adhesive, and silanization fluid were applied according to the manufacturers' instructions (Clearfil SA primer, Clearfil Photobond and Activator, Kuraray, Osaka, Japan).

The specimens of both groups were restored with a heavily filled hybrid resin composite material (Clearfil Photo Posterior US, Kuraray, Osaka, Japan). This material was also used for the original restorations, following the same restorative procedure, including a polyvinylsiloxane mould to copy a standard external shape to all the restorations to be constructed [20]. The mould consisted of three parts in order to enable build-up of the repaired restoration in layers of 2 mm composite material maximum, using an injection technique. Each layer was light-cured for 40 seconds (Translux CL, Heraeus Kulzer, Hanau, Germany). Intensity of the light-curing unit was 420 mW/cm^2^ as measured before and after the experiment using a curing radiometer. After repair the specimens were finished using polishing discs (Sof-Lex, 3M Espe, St Paul, MN, USA).

### 2.3. Load Test

The conditions of the load test were identical to the conditions in the previous study [20]. The specimens were mounted in a material testing machine (Lloyd LRX, Lloyd Instruments, Fareham, UK) with the occusal surface horizontally, and a vertical static load was applied. Crosshead speed of the testing machine was 0.5 mm/min. The load was applied with a 4 mm diameter stainless steel cylinder in the central occlusal groove that loaded both cusps halfway the buccal and palatal slope [19–21]. Load until fracture was registered for each specimen. After fracture, the failure mode was categorized on a visual basis, and a distinction was made between (1) intact tooth substrate (fracture along repaired surface or fracture along repaired surface with new fracture surfaces into resin composite) and (2) fractured tooth substrate (fracture of tooth fragments below cementoenamel junction (CEJ) or vertical root fracture). Classification was based on a two-examiner agreement.

### 2.4. Statistical Analysis

For the comparison of the fracture loads a two-tailed *t*-test was used. Fracture loads of the repaired restorations and the original restorations were compared using paired *t*-tests. The differences in failure modes between repaired woven and unidirectional FRC were analyzed by using a Fisher's exact test. All tests were performed at a significance level of 5% with SPSS, version 15.0 (SPSS Inc., Chicago, Ill, USA).

## 3. Results

**Figure 3:**
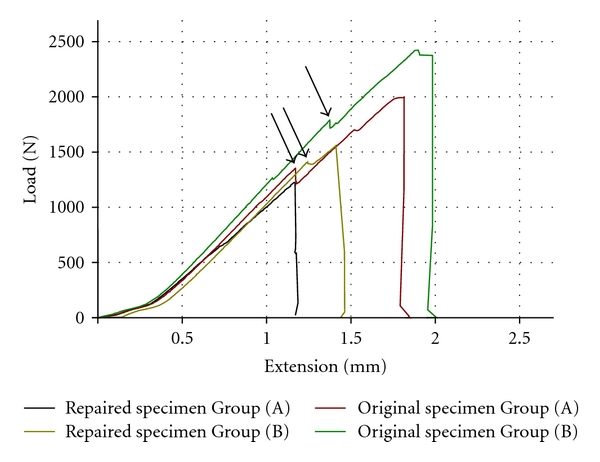
Representative fracture graphs from the repaired and the original specimens (arrows indicate initial failure).

Repaired specimens fractured at failure loads of 820 N up to 1559 N (Table 2). The differences in fracture loads between the repaired Groups (A) and (B) were not statistically significant (*P* = 0.34). Furthermore, the fracture loads of the repaired specimens in this study were significantly lower than those of the original specimens (*P* = 0.02 for Group (A) and *P* < 0.001 for Group (B)). Representative fracture graphs from the repaired and the original specimens are shown in Figure 3. The failure of the repaired specimen in Group (A) in particular was more sudden when compared to the other three graphs, which showed a preceding initial failure.

**Figure 4:**
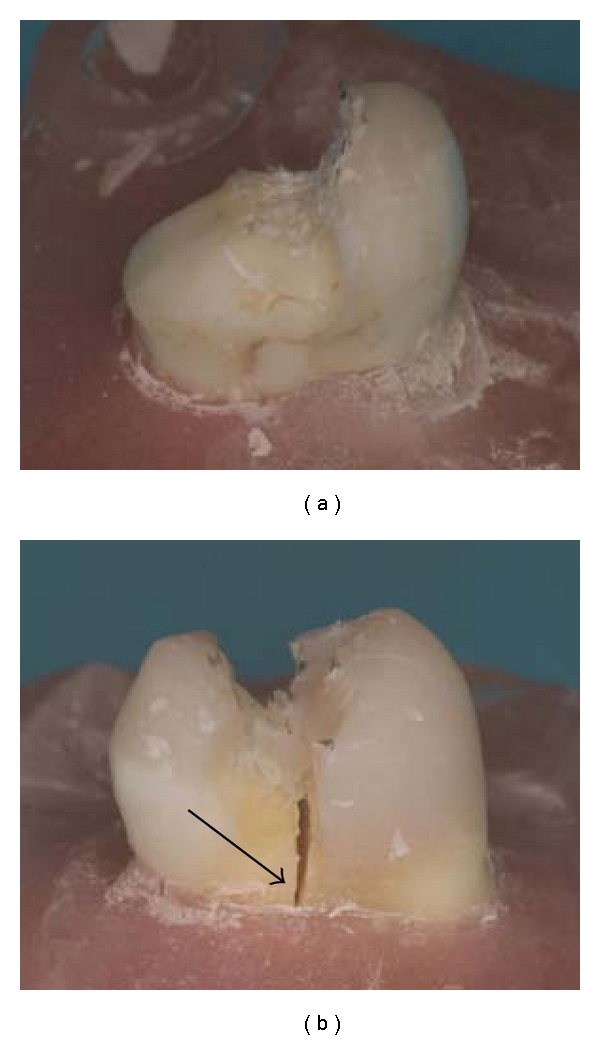
Examples of repaired specimens with woven FRC basing after the load test; fracture along the repaired surface (a) and vertical root fracture, indicated by the arrow (b).

Different failure modes were observed (Table 3 and Figure 4). The majority of specimens showed failure along the repaired surface. Furthermore, all but one specimen in repaired Group (B) (89%) showed fracture of the restoration without fracture of tooth substrate (Table 3). In repaired Group (A) such an intact tooth substrate after restoration fracture was observed in two cases (28%). This difference in proportions was statistically significant (*P* = 0.04).

Of the specimens with intact tooth substrate, new fracture surfaces developed into the resin composite (Table 3). This applies for one out of two specimens in repaired Group (A), which had lost the resin composite material covering the palatal cusp, and six out of eight specimens in repaired Group (B), for which fracture of the new resin composite material in the approximal boxes occurred.

In case of fracture of tooth substrate, fracture of a tooth fragment was combined with fracture along the repaired surface (Table 3). The vertical root fractures in repaired Group (A) did not show adhesive failure along the repaired surface, but cohesive fracture of the resin composite material in the central part of the restoration.

## 4. Discussion

An attractive feature of resin materials is their potential of repair, although there are difficulties in adhering new resins to the previously polymerized and aged light-cured composites. Clinical study of repaired restorations meets problems regarding the type and characteristics of the fractured restorations that have to be repaired. Uniformity of specimens is nearly impossible as is the making of an estimation of the expected fail period. A preliminary step into this clinical question is to use fractured specimens in laboratory research. In this study, the specimens originated from a static loading experiment and were stored for a longer time in a humid environment, as to imitate ageing. To our knowledge this has not been done yet. The positive side effect of this approach is that we did not need to simulate fracture by cutting the cusp-replacing FRC restorations. Before repair, the restorations showed a genuine exposed surface by fracture including internal microcracks and other surface irregularities. In line with a clinical preservative treatment approach, the strategy was to do the minimum, which is just adding resin composite without application of additional FRC layers. The minimum intervention also applied to the adhesive procedure. We used a silane coupling agent which promotes adhesion between resin and hydroxyl group covered glass fibers but does not affect adhesion with the polymer matrix. An alternative approach could have been to utilize a secondary interpenetrating polymer network (IPN) bonding mechanism. This was, however, not applied given the practical limitations of the required impregnation time of uncured bonding in the clinical situation. With regard to the design of the load test, a next step can be to apply dynamic loading. An advantage of the static loading in the present study was, however, that fracture loads of the repaired specimens could be compared to those of the original specimens.

The effect of the long water storage on the fracture resistance of FRC restorations is not fully understood. It has been shown that flexural strength of FRC decreases due to water sorption [23]. Another study reported, however, that water immersion of FRC did not significantly affect impact strength [24]. It is anticipated that surface hydrolyses affect bonding capacity and exposure to water is clinically relevant if the patient returns to the dental office with a time lap after fracture. Yet, the time dependency of the water exposure and bonding capacity is not clear.

The fracture loads in the present study were about 50% lower than the fracture loads of the original specimens (Table 2). In the literature there is agreement that the adhesive strength of a repair is substantially lower than the cohesive strength of the material, but there is debate about the rate of decrease. Reported decrease of cohesive strengths of repaired resin composite materials varies from 2% to 69% [1, 3, 5]. For FRC in particular a decrease of 15% to 30% after repair was reported [3, 5]. If the fiber layer was damaged the decrease was more pronounced, being about 50% [5]. The latter condition is in accordance with the present restorations since several of the fiber layers were also partially disrupted. Before repair, specimens with woven fibers showed more severe damage than specimens with unidirectional fibers (Table 1). The damaged fiber layer may explain the straight curve in the fracture graph for the repaired specimen in Group (A) (Figure 3), showing similar behavior to particulate resin composite. This is in contrast with the other groups that showed initial failure before final failure, which is the natural behavior of FRC materials.

The basic layer of the original restoration included unidirectional or woven preimpregnated glass fibers. Testing the repaired restorations, no difference was observed between fracture loads with either type of fiber reinforcement. This matched with the loading results of the original restorations in the previous study. Recently it was reported that shear bond strength of resin composite to fiber-reinforced substrates depends on the load to fiber direction. For the load direction that corresponds to the direction of the fibers shear resistance was higher than for the load direction perpendicular to the fibers [25]. In our study the unidirectional fibers had a buccal-palatal orientation in order to prevent separation of the cusps under occlusal loading. With the applied load, stress had the same direction, but we did not find the above difference. Possibly the complex cavity geometry caused compressive and tensile forces at the interface as well.

With regard to the failure mode, adhesive fracture along the repaired surfaces could be expected, without fracture of tooth substrate, since bonding new to old composite goes with a decrease of strength. This was partly seen. Sixty-three percent of the specimens (10 cases) fractured mainly along the repaired surface without involvement of tooth substrate. These adhesive failures were combined with cohesive fracture of the resin composite material in the cavity boxes for a majority of specimens, which may be due to the macromechanical resistance in these areas. Failures with fracture of tooth substrate were more frequently observed with woven FRC than with unidirectional FRC, while this difference was absent in the previous loading study [20]. The partial damaging of fiber layers during the previous load test may explain the insufficient protection of the underlying tooth substrate. The more severe damage of specimens with woven fibers adds to the present result, which suggests that application of additional FRC may be beneficial. Additionally, specimens without visible fracture of tooth substrate were used, but undetected internal cracks as a result of the previous load test might be the origin of fracture of tooth substrate. Of the specimens with fracture of a tooth fragment, an adhesive fracture preceded the loss of dentine below the CEJ. Vertical root fracture was accompanied with cohesive fracture of the resin composite, which was in accordance with the results of previous studies [19–21]. This suggests that cohesive fracture of the restoration is a sign of a destructive failure, likely to propagate deep into the tooth.

The decrease in fracture resistance when compared to the original cusp-replacing restorations suggests that repair includes a risk of repeated fracture, especially since failure at the repaired interface was frequently observed. Although one can doubt the validity of loading values found in laboratory tests, values of 1200 N seem acceptable for application in clinical repairs.

## 5. Conclusion

Cusp-replacing resin composite restorations with an FRC basing that are repaired with a resin composite show about half of the fracture resistance under loading compared to the original restorations. The mode of failure with a base of unidirectional fibers is predominantly of an adhesive nature.

## Figures and Tables

**Table 1:** Condition of the specimens before repair.

Group	Restoration zone	Fiber layer present		Fiber layer and resin composite absent (%)
		Resin composite present (%)	Resin composite absent (%)	
(A) Restorations with woven FRC basing (*n* = 7)	Palatal cusp^(1)^	100	0	0
	Step^(2)^	0	100^(5)^	0
	Mesial/distal box^(3)^	7	50^(5)^	43
	Buccal cusp^(4)^	0	48^(6)^	52

(B) Restorations with unidirectional FRC basing (*n* = 9)	Palatal cusp^(1)^	93	0	7
	Step^(2)^	11	89	0
	Mesial/distal box^(3)^	17	50^(7)^	33
	Buccal cusp^(4)^	0	74^(8)^	26

^(1)^Corresponds with areas 1, 2, and 3 of Figure 2.

^(2)^Corresponds with area 5 of Figure 2.

^(3)^Corresponds with areas 4 and 6 of Figure 2.

^(4)^Corresponds with areas 7, 8, and 9 of Figure 2.

^(5)^43% loose fiber layers.

^(6)^20% loose fiber layers.

^(7)^11% loose fiber layers.

^(8)^35% loose fiber layers.

**Table 2:** Fracture loads of repaired specimens in newton.*

Group				95% CI for mean	
	*N*	Mean	SD	Lower bound	Upper bound
(A) Woven FRC basing	7	1261	228	1017	1505
(B) Unidirectional FRC basing	9	1127	242	920	1335

*Data original restorations: woven FRC basing 2202 (SD 200 N), unidirectional FRC basing 2426 (SD 333 N).

CI: confidence interval; SD: standard deviation.

**Table 3:** Condition of the repaired specimens after the load test.

Group	Intact tooth substrate*		Fractured tooth substrate*	
	Fracture along repaired surface	Fracture along repaired surface with new fracture surfaces	Fracture of tooth fragment below CEJ	Vertical root fracture
(A) Woven FRC basing (*n* = 7)	1	1^(1)^	2	3
(B) Unidirectional FRC basing (*n* = 9)	2	6^(2)^	1	0

*Difference in proportions between repaired Groups (A) and (B) statistically significant (Fisher's exact test, *P* = 0.04).

^(1)^Resin composite material covering palatal cusp not present anymore.

^(2)^Resin composite material left in mesial and/or distal box.

CEJ: cementoenamel junction.
